# Transfer of piano practice in fast performance of skilled finger movements

**DOI:** 10.1186/1471-2202-14-133

**Published:** 2013-11-01

**Authors:** Shinichi Furuya, Ayumi Nakamura, Noriko Nagata

**Affiliations:** 1 Institute for Music Physiology and Musicians’ Medicine, Hannover University of Music, Drama and Media, Emmichplatz 1, Hannover 30175, Germany; 2 Graduate School of Science and Technology, Kwansei Gakuin University, Sanda, Japan

**Keywords:** Transfer, Motor learning, Neuroplasticity, Fine motor control, Motor skill

## Abstract

**Background:**

Transfer of learning facilitates the efficient mastery of various skills without practicing all possible sensory-motor repertoires. The present study assessed whether motor practice at a submaximal speed, which is typical in sports and music performance, results in an increase in a maximum speed of finger movements of trained and untrained skills.

**Results:**

Piano practice of sequential finger movements at a submaximal speed over days progressively increased the maximum speed of trained movements. This increased maximum speed of finger movements was maintained two months after the practice. The learning transferred within the hand to some extent, but not across the hands.

**Conclusions:**

The present study confirmed facilitation of fast finger movements following a piano practice at a submaximal speed. In addition, the findings indicated the intra-manual transfer effects of piano practice on the maximum speed of skilled finger movements.

## Background

One key element of skillful behaviors resides in production of various sensory-motor repertoires. Acquisition of diverse skills does not necessarily require practicing all possible movement and perceptual repertoires. A neural mechanism that facilitates untrained skills via practicing a certain skill, so called “transfer of learning”, enables the efficient mastery of various sensory-motor skills. Understanding the learning transfer therefore sheds light on the mechanisms underlying the skill acquisition.

In sports and musical performance, the acquisition of a novel motor skill typically begins with practicing at movement speed substantially slower than the maximum speed. This not only allows the body to be moved along the desired trajectory via feedback control, but also provides the learner with sensory feedback to update movement planning and execution [1]. Consequently, movements become more efficient and accurate at the practiced speed [2], which may enable the learner to challenge faster movements. Yet, it is unclear how motor practice at a submaximal speed influences the rapid performance of movements at trained and untrained tasks and effectors, which is a representative goal of skillful motor behaviors [3-5].

Studies have extensively examined neuroplasticity subserving motor skill learning. These studies have revealed structural and functional changes in the cortical and subcortical regions responsible for fast and accurate sequential movements [6,7]. For example, neuroimaging studies have demonstrated that the enlargement of motor-related cortical areas through long-term musical training was associated with the facilitation of the maximum speed of finger movements [8,9]. In addition, extensive practice of a complex finger movement as fast and accurately as possible elicited more activation in the motor regions and faster finger movements [10]. These findings suggest that an increased maximum speed of finger movements requires larger neural resources. Because finger movements at a submaximal speed use only a small portion of the motor-related cortical regions [11,12], it is unlikely that motor practice at a submaximal speed results in an increase in the maximum speed of finger movements that activates large regions. A decrease of neural activation in the motor areas while moving the fingers at a particular speed due to extensive piano practice [13] presents the alternative possibility that piano practice *per se* economizes the neural activity responsible for skillful finger movements and thereby provides additional cortical resources to move the fingers faster. However, whether training of finger movements at a submaximal speed increases the maximum movement speed has not been tested by previous studies on the skill learning of sequential finger movements, which identified improvements in speed and accuracy after practicing as fast and accurately as possible [14] and improvements in accuracy after practicing at a certain movement rate [15]. Furthermore, transfer effects of the training across sequences and hands have not been well-addressed. Evidence for shared movement elements across various motor repertoires [4,16,17] postulates transfer of learning across motor sequences. By contrast, there has been no converging evidence about the inter-manual transfer of learning of skilled finger movements [14,18-20]. Using daily piano practice, the present study assessed whether motor practice at a submaximal speed yields an increase in the maximum speed of both trained and untrained fast skilled finger movements at the trained and untrained hand.

## Methods

Six musically naïve young male individuals (21.3 ± 1.8 yr) (“training group”) and six age-, gender-matched musically naïve individuals (20.9 ± 2.5 yr) (“control group”) participated in the study. All participants were right-handed with the laterality index of 89.6 ± 8.9 (all >80) [21]. None of the participants had played any musical instruments before the experiment. The experimental protocol was approved by the local ethics board of Kwansei Gakuin University, and all participants gave informed consent prior to the experiment. The experiment was carried out according to the Declaration of Helsinki.

**Figure 1:**
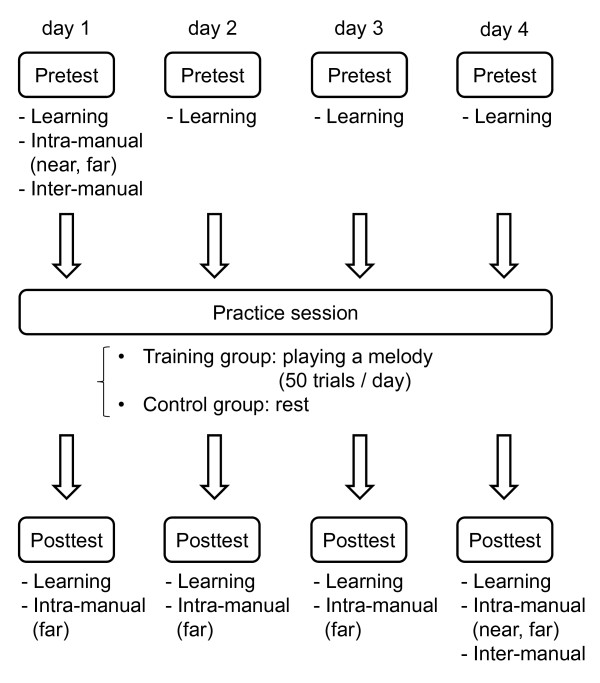
A design of the current study with experiments over four successive days.

**Figure 2:**
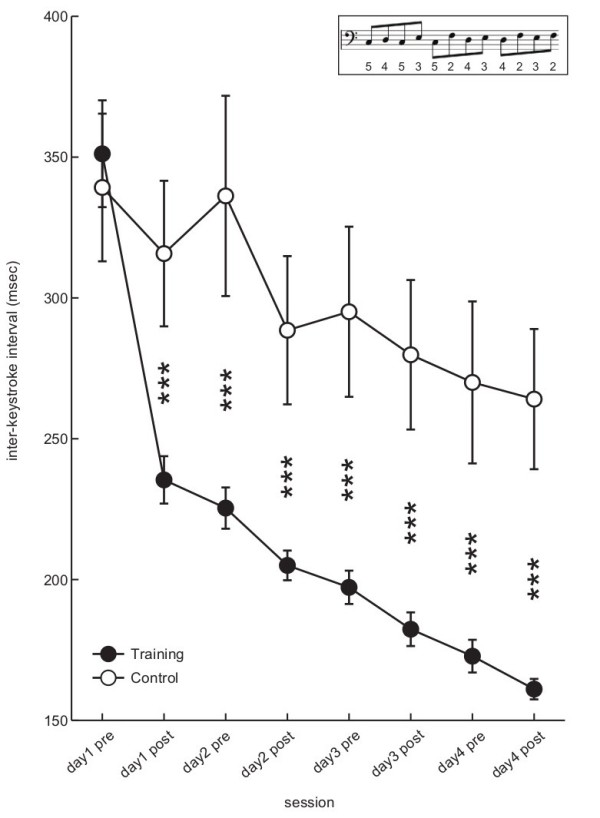
**The group mean of the inter-keystroke interval (IKI) while playing a practiced tone sequence as fast and accurately as possible over four successive days of the sessions for the trained group (filled circle) and control group (open circle).** An error bar indicates the standard error. ***: p < 0.001. (Subsets) a tone sequence used for the current practice and learning test. The numbers indicate fingering (2 to 5 correspond to index to little finger).

The experiment consisted of a practice session for four successive days (only for the training group) and pre- and post- test sessions prior to and following the practice session (for both two groups) (Figure 1). During the practice session, each participant of the training group played a certain tone sequence consisting of twelve strokes with a predetermined fingering that used all possible pairs of fingers (Figure 2, subset) with the left hand. We chose the non-dominant left hand because this hand is less frequently used in daily and sports activities compared with the dominant hand. The participant played a digital piano (YAMAHA, P-250) with an inter-keystroke interval (IKI) of 500 ms in synchronization with a metronome (two strokes per second) at a predetermined loudness (90 MIDI velocity). The score with fingering was displayed on a monitor in front of each participant. This task was repeated 50 times per day in the training group, and trials that included erroneous stroke(s) and/or stronger or softer stroke(s) (±5 MIDI velocity) were repeated. An erroneous trial was identified based on MIDI information displayed on a PC monitor in front of the experimenter during data collection, and the total number of erroneous trials was not counted. The whole sessions were not video-taped in the present experiment. Prior to the data recording on the first day, each participant in both of the training and control groups was allowed to practice to familiarize themselves with both the given tone sequence and the piano by accepting instructions from the experimenter, which took approximately five minutes. During the familiarization session, the experimenter supervised which keys were to be struck with which fingers without providing any explicit instructions about how to move fingers. All participants memorized the sequence during the familiarization session, and throughout the subsequent experiments with data collection, we rarely observed erroneous keystrokes. Each participant in the control group took a rest for 20 minutes between the pre-test and post-test of each day, which approximately corresponds to the duration of 50 trials in the training group.

**Figure 3:**
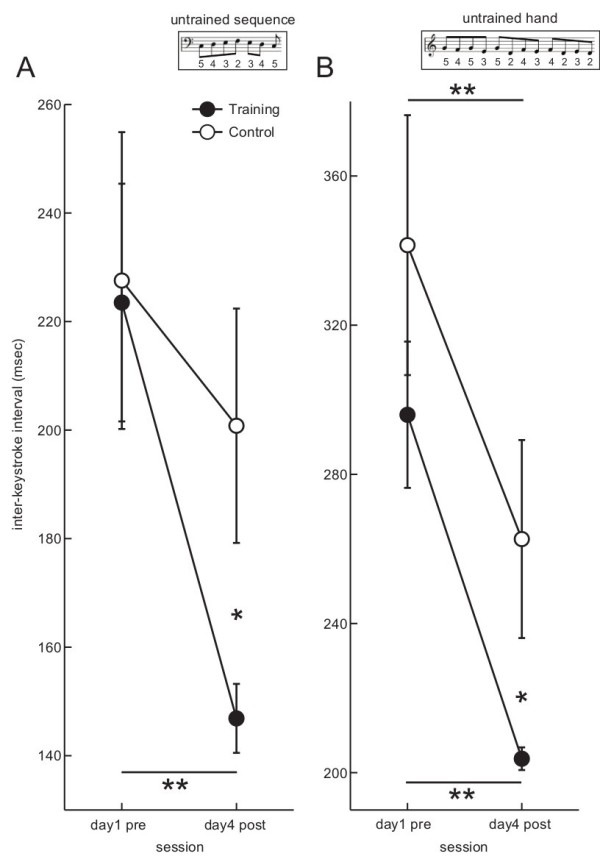
**The group mean of the inter-keystroke interval (IKI) while playing an unpracticed tone sequence as fast and accurately as possible (A), and a mirrored tone sequence with an unpracticed right hand (B) at the first and final days of the practice sessions for the trained group (filled circle) and control group (open circle).** An error bar indicates the standard error. *: p < 0.05, **: p < 0.01. (Subsets) a tone sequence used for the current intra-manual near transfer test **(A)**, and the inter-manual transfer test **(B)**. The numbers indicate fingering (2 to 5 correspond to index to little finger).

During the pre- and post-test sessions, each participant was asked to perform several motor tests to evaluate effects of the practice on the trained and untrained motor skills. These included (1) playing the practiced tone sequence as fast and accurately as possible (learning test), (2) playing an unpracticed tone sequence that is similar to the trained sequence as fast and accurately as possible (intra-manual *near* transfer test, Figure 3A subset), (3) striking a piano key repetitively with each of four fingers as fast and accurately as possible while the remaining digits depressed adjacent keys (intra-manual *far* transfer test) [22], and (4) playing a mirror-image sequence with the same fingering used for the practiced left-hand sequence with the right hand as fast and accurately as possible (inter-manual transfer test, Figure 3B subset). The intra-manual *far* transfer test was performed for six seconds before the practice session on the first day and after the practice session on all four days to minimize the muscular fatigue elicited by this task. Both the intra-manual *near* and inter-manual transfer tests were conducted prior to the practice session on the first day and following the practice session on the fourth day to minimize participants’ familiarity with these tasks.

During the experiment, MIDI (Musical Instrument Digital Interface) data that include information on pitch, loudness, and timing of keystroke and key-release were collected from the piano using a custom-made script written in JAVA with a time resolution of one millisecond. This allowed us to record the time each key was depressed and the time it was released, and to compute the IKI as an interval from key depression to key depression, which was averaged within a trial.

To statistically evaluate the effects of daily piano practice on the motor functions of fingers, we used a two-way mixed-design analysis of variance (ANOVA) with “session” (within variable) and “group” (between variable) as the independent variables, with Tukey post hoc tests with multiple comparisons. We used an eta-squared (η^2^) measure as an index of effect size. We predicted the interaction effect of these variables, being larger changes in the movement variables over sessions for the training group than the control group. Statistical analysis was performed by using R (ver. 3.0.0).

## Results

Figure 2 displays the group mean of the within-trial average of the IKI while playing the practiced tone sequence as fast and accurately as possible (learning test) over four successive days for the training and control groups. First, the IKI during the pre-test of the first day (i.e. before starting the practice) was smaller than the IKI used for the practice (=500 ms) for all participants (two groups pooled, 345.2 ± 53.8 ms), which was also statistically significant (one-tailed t-test, p = 3.8 × 10^-7^, r = 0.95, t = −9.97, df = 11). The findings confirmed that the current practice was performed at tempo sufficiently slower than the fastest tempo. Second, the practice group displayed a larger decrease in the IKI over sessions than the control group, which confirmed that the current slow practice facilitated the maximum speed of successive keystrokes. The maximum speed doubled after the four days of practice. A two-way mixed-design ANOVA confirmed a significant interaction effect of session and group (F(7,70) = 13.7, p = 5.0 × 10^-11^, eta-squared = 0.13), and main effects of session (F(7,70) = 64.8, p = 4.6 × 10^-28^, eta-squared = 0.42) and group (F(1,10) = 8.7, p = 0.01, eta-squared = 0.44). Tukey’s post-hoc tests revealed a significant group difference at each of the sessions except for the pre-test of the first day (Figure 2). The results indicate that the training group benefited from the current practice, although the pre-test and post-test themselves also facilitated the fast finger movements for both of the two groups.

In order to further evaluate the retention of the slow practice effect on the trained motor skill, each participant in the training group was again asked to perform the above learning test two months after the fourth day of the practice session. During the intervening two months, each participant was asked not to play the piano at all. The mean and standard deviation of the IKI was 195.7 ± 25.4 ms, and a paired t-test did not yield a significant difference between the post-test of the fourth day of the practice session and two months after it (p = 0.14, r = 0.61, t = −1.73, df = 5), confirming the retention of the trained motor skill.

Figure 3 shows the group mean of the within-trial average of the IKI while playing an unpracticed tone sequence with the left hand as fast and accurately as possible (A, intra-manual near transfer test) and while playing a mirror-image tone sequence with the contra-lateral right hand as fast and accurately as possible (B, inter-manual transfer test) for the training and control groups. The maximum speed while playing an unpracticed sequence became 1.5 times faster after the four days of slow practice. A two-way mixed-design ANOVA identified a significant interaction effect between session and group (F(1,10) = 5.2, p = 0.04, eta-squared = 0.09), confirming an intra-manual transfer effect of the current practice. Also, a post-hoc identified a group difference only at the post-test of the fourth day (p = 0.04). A main effect of session (F(1,10) = 19.6, p = 0.001, eta-squared = 0.23) but not of group (F(1,10) = 1.2, p = 0.31, eta-squared = 0.09) was significant. The maximum speed of the unpracticed right hand also became faster following practice at the both groups following the four days of sessions. A two-way mixed-design ANOVA failed to confirm a significant interaction effect (F(1,10) = 0.3, p = 0.60, eta-squared = 0.01), although a post-hoc test identified a group difference only at the post-test of the fourth day (p = 0.03). A main effect of session (F(1,10) = 88.0, p = 2.8 × 10^-6^, eta-squared = 0.47) but not of group (F(1,10) = 0.2, p = 0.65, eta-squared = 0.02) was significant. To directly assess a difference in the intra-manual and inter-manual transfer effect, a three-way mixed-design ANOVA using session, group, and test-type (i.e. intra-manual or inter-manual) was performed. A significant interaction effect between session and test-type was evident (F(1,10) = 6.5, p = 0.03, eta-squared = 0.05), indicating that the intra-manual transfer effect was more pronounced than the inter-manual transfer effect. The result suggests that benefit of practice on performance speed transfers more across sequences than across effectors.

In order to evaluate the retention of the observed intra-manual *near* transfer effect, the retention test was performed two months after the sessions. The mean and standard deviation of the IKI during the intra-manual *near* transfer test was 154.9 ± 19.8 ms, and a paired t-test did not yield a significant difference between the post-test of the fourth day of the session and two months after it (p = 0.13, r = 0.63, t = 1.82, df = 5), confirming the retention of the intra-manual *near* transfer effect.

As a supplementary analysis to assess whether the individuated finger movements improved following the current practice, the participants performed the fastest keystrokes with each of four fingers while the remaining fingers continued to depress the adjacent keys (intra-manual far transfer test). Table 1 illustrates the group mean of the within-trial average of the IKI during the single finger tapping over four successive days for the practice and control groups, and results of ANOVA. All four fingers displayed faster keystroke rates over session for both groups. A three-way mixed-design ANOVA using session, group, and finger as independent variables revealed neither significant interaction effect nor main effect of group (Table 1), which failed to confirm a larger improvement of the individuated finger movements over session for the training group than the control group.

**Table 1:** Results of the fastest tapping of each of the four fingers

		**Group mean and SD of the inter-keystroke interval (ms)**									
		**session**									
		**Day 1 pre**		**Day 1 post**		**Day 2 post**		**Day 3 post**		**Day 4 post**	
I	Tra	204.8	(21.1)	198.5	(26.0)	194.8	(14.3)	190.5	(16.8)	187.6	(22.9)
	Ctl	240.6	(56.9)	222.7	(38.5)	208.6	(30.7)	216.8	(27.0)	212.4	(23.5)
M	Tra	232.8	(28.2)	223.8	(30.5)	232.1	(23.8)	219.9	(30.8)	213.3	(28.3)
	Ctl	250.1	(45.3)	241.9	(29.0)	230.5	(35.3)	231.5	(33.8)	223.4	(26.2)
R	Tra	271.5	(51.8)	248.0	(37.4)	266.4	(31.8)	243.1	(32.2)	232.2	(24.8)
	Ctl	334.0	(92.2)	301.8	(58.1)	292.2	(42.3)	276.7	(37.0)	258.9	(23.2)
L	Tra	251.8	(35.8)	232.3	(35.3)	239.5	(25.3)	220.4	(29.7)	219.9	(28.7)
	Ctl	267.0	(36.7)	257.5	(27.2)	258.6	(24.6)	253.6	(22.7)	246.8	(27.8)
ANOVA results											
		group	session	finger	group x	group x	session x	group x finger x session			
					finger	session	finger				
dof		1,10	4,40	3,30	3,30	4,40	12, 120	12, 120			
F		2.1	10.7	55.6	2.7	0.97	3.35	1.38			
p		0.18	<0.001	<0.001	0.06	0.43	<0.001	0.18			
η2		0.13	0.35	0.1	0.03	0.01	0.03	0.01			

I, M, R, L indicates the index, middle, ring and little finger, respectively.

Tra: training group, Ctl: control group.

A number in parenthesis indicates standard deviation within each of the training and control groups.

η2 indicates eta-squared.

To assess the effects of the practice on rhythmic accuracy of the keystrokes, the error of IKI (i.e. value between the played and target IKI) was computed for each stroke and averaged within a trial, and then averaged across the first five and the final five trials of each practice session separately. A two-way mixed-design ANOVA yielded neither interaction effect of group and session (F(7,70) = 1.07, p = 0.39, eta-squared = 0.03) nor significant main effects of group (F(1,10) = 0.24, p = 0.63, eta-squared = 0.02) and session (F(7,70) = 1.07, p = 0.39, eta-squared = 0.03), which confirmed a lack of improvement in rhythmic accuracy after the current slow practice.

## Discussion

This study demonstrated an increase in the maximum speed of sequential finger movements after musically naïve individuals practiced a short sequence of piano keystrokes at a submaximal tempo. The increase was not due to the speed-accuracy tradeoff [23,24], because the movement accuracy did not decrease with the current practice. In addition, the improvement of the maximum speed of the skilled finger movements is unlikely to result from familiarization to the task due to a substantially larger increase in the movement speed for the trained group than the control group. A novelty of the present findings is an increase in the maximum speed of sequential finger movements through practicing at a submaximal speed, which has not been addressed in previous studies that investigated effects of practicing as fast and accurately as possible [10,14]. It is thus likely that facilitation of fast finger movements does not necessarily require practicing at the maximal speed.

Enhancement of the speed of finger movements has been previously reported following extensive training of complex finger movements as fast and accurately as possible, which increases the cortical activity responsible for the finger movements [10]. Individuals with extensive piano training can also perform complex finger movements at a particular speed with less activation of the motor-related regions [13]. It is thus possible that the practice *per se* economized the neural activity responsible for skilled finger movements and thereby provides additional cortical resources to move the fingers more rapidly.

Enhancement of fast finger movements was not limited to the practiced sequence of movements. The maximum movement speed was also enhanced while performing an unpracticed sequence of finger movements similar to the trained sequence. However, the single finger tapping, which differs considerably from the trained task, displayed no practice-specific improvement. It is thus unlikely that the slow practice facilitated independent control of finger movements [25,26], and thereby enhanced fast sequential finger movements. These findings indicate that the intra-manual transfer of the slow practice depends on similarity of motor skills to the trained task, which corroborates the concept of similarity-dependent transfer of learning [20,27]. The intra-manual transfer-of-learning in sequential finger movements can be related to fundamental motor patterns shared across sequences [16,22,28]. Practicing a particular motor sequence may therefore facilitate the fundamental motor skill, and thereby influence similar but even untrained sequential movements. Future studies using neurophysiological evaluations are needed to elucidate the underlying neural mechanisms.

The enhancing effect of the current motor sequence practice did not generalize to the untrained contra-lateral right hand. This finding corroborates the previous finding of no inter-manual transfer effect of practicing a sequence of finger movements as fast and accurately as possible [14,29]. However, some studies showed that unimanual practice of the fast finger movements improved motor skill of the untrained hand [18,19,30]. These differences across studies may suggest that the inter-manual transfer of motor skill learning depends on nature of motor practice. It is also possible that the inter-manual transfer depends more on time than on practice.

Remarkably, the facilitated motor functions of the fingers were evident even two months after the final practice session. This retention effect, which is typically observed when learning sequential finger movements [31], suggests long-term impacts of daily piano practice on motor skills responsible rapid sequential finger movements.

Rhythmic accuracy of the sequential finger movements did not improve following the current slow practice. This finding was inconsistent with previous findings that identified a slight increase in the temporal accuracy of sequential finger movements while playing music after short-term piano practice [15,32]. The difference may be attributed to the varying difficulty of the task performed by players across studies. Although previous studies identified a decrease in the number of erroneous keystrokes with practice, the participants in the present study produced almost no erroneous keystrokes even on the first recording trial, presumably because feedback control mediated their slow movements [33].

An open question is whether the present practice has the same impact on highly-skilled individuals. Several studies of perceptual and motor learning demonstrated that trained individuals benefited more from training [34]. For example, musicians showed a larger training-related improvement of the tactile discrimination ability compared with non-musicians [35]. By contrast, a recent study using a transcranial direct current stimulation revealed a smaller improvement of fine motor control after behavioral training with the stimulation for pianists who commenced musical training at earlier age [36]. A further study is needed whether the current piano practice yields larger or smaller facilitation of fast finger movements in trained musicians.

## Conclusions

The present study demonstrated an increase in the maximum speed of skilled finger movements at the trained hand after practicing at a submaximal speed. The facilitation of movement speed at both trained and untrained sequences provided evidence supporting for the intra-manual transfer of motor practice in skilled finger movements. However, the facilitation was not observed between the hands, suggesting an effector-specific benefit of motor sequence practice on subsequent speed of motor sequence performance. The finding implicates a potential of the slow practice as an intervention of focal hand dystonia, which exacerbates fine motor control particularly when moving fast [37,38].

## Competing interests

The authors declare no disclosure of financial interests and potential competing interests.

## Authors’ contributions

SF participated in the design of the study, analyzed data, performed the statistical analysis, and drafted the manuscript. AN performed the experiment and analyzed data. NN participated in the design and coordination of the study and helped to draft the manuscript. All authors read and approved the final manuscript.
